# Rhizobial Inoculation Improves Soil Properties and Microbial Network Stability to Support *Medicago sativa* L. Production in Cold Arid Regions

**DOI:** 10.3390/microorganisms14071427

**Published:** 2026-06-30

**Authors:** Qianqian Zhao, Xin Jin, Chengti Xu, Guangxin Lu, Haijuan Zhang

**Affiliations:** 1 College of Forestry and Grassland, Qinghai University, Xining 810016, China; 13141752025@163.com (Q.Z.); 18894310895@163.com (X.J.); 2 Northwest Key Laboratory of Cultivated Land Conservation and Marginal Land Improvement, Ministry of Agriculture and Rural Affairs, Delingha 817000, China; xchti@163.com; 3 College of Animal Husbandry and Veterinary Sciences, Qinghai University, Xining 810003, China; 4 National Grass Varieties Regional Test Station, Delingha 817000, China; 5 Qinghai Provincial Key Laboratory of Plateau Climate Change and Corresponding Ecological and Environmental Effects, Qinghai Institute of Technology, Xining 810016, China

**Keywords:** Qaidam Basin, *Medicago sativa* L., rhizobial inoculant, rhizosphere soil bacteria, microbial network stability

## Abstract

The Qinghai–Tibet Plateau, a globally significant ecological barrier and a core pastoral region, is persistently constrained by cold and arid climatic conditions, nutrient poor soils, and progressive grassland degradation. These challenges necessitate maintaining forage productivity while enhancing ecological stability. *Medicago sativa* L., valued for its high nutritional quality and capacity for biological nitrogen fixation, has been widely incorporated into regional grassland systems. Rhizobial inoculation, as an environmentally sustainable agronomic practice, is regarded as an effective approach to improving nutrient use efficiency and promoting ecological restoration; however, its underlying mechanisms in cold and arid environments remain insufficiently understood. This study established a field experiment in Delingha, Qaidam Basin, using the cultivar ‘Beilin 201’. Treatments included an uninoculated control (CK) and four rhizobial seed coating rates: E1 (0.75 g·m^−2^), E2 (1.50 g·m^−2^), E3 (2.24 g·m^−2^), and E4 (3.00 g·m^−2^). The effects on yield, rhizosphere soil physicochemical properties, bacterial community structure, and molecular ecological networks were systematically evaluated. The composite microbial inoculant maintained *Medicago sativa* L. yield, with only modest and non-significant increases in some treatments. In contrast, soil organic matter increased significantly with application rate (*p* < 0.001), suggesting a stronger short-term effect on soil properties than on yield. Although network vulnerability was lowest in E4, the differences among treatments were not statistically significant. Mixed effects modeling showed that soil factors (74.79%) and microbial factors (25.12%) jointly influenced yield variation. Structural equation modeling further revealed that microbial factors exerted a positive direct effect on yield (0.3), whereas soil factors exhibited a stronger direct effect (0.57), with inoculation rate primarily influencing yield indirectly through soil mediated pathways. This study elucidates the ecological functions and regulatory mechanisms of rhizobial formulations in high elevation dryland ecosystems and provides both theoretical support and practical guidance for the rational application of microbial fertilizers and the sustainable management of forage systems on the Qinghai–Tibet Plateau.

## 1. Introduction

At the global scale, grassland degradation is widely recognized as an urgent threat to the sustainability of grassland agriculture and livestock production. Under the compounded pressures of climate change and human disturbance, productivity and biodiversity continue to decline, exacerbating forage deficits and the erosion of ecosystem services [1,2,3]. On the Qinghai–Tibet Plateau, cold and arid climates, along with fragile ecosystems, amplify these stresses; despite multiple restoration programs, the overall trend has not been fundamentally reversed [4]. High-elevation inland basins, such as the Qaidam Basin, are characterized by intense evapotranspiration, scarce precipitation, and pronounced spatial heterogeneity. Long-term observations indicate that the Delingha region is particularly sensitive to warming and moisture variability, thereby narrowing the window for harvesting high-quality forage and intensifying feed-livestock conflicts [5,6,7,8]. Moreover, soil microbial diversity is highly responsive to elevation, salinity, and hydrothermal regimes, which constrain the stability of production systems [9,10]. Against this backdrop, boosting the production of high-quality forage and enhancing soil functions has become an urgent priority.

In response, *Medicago sativa* L., renowned as the “queen of forages” for its high protein content, excellent digestibility, and broad stress tolerance, has long played a central role in ruminant husbandry and grassland agriculture [11,12,13,14]. Its crude protein content is typically reported at 15–22%, strongly modulated by genotype, ecoregion, and harvest stage [15,16,17], while its yield and quality are co-shaped by environmental and managerial factors [18]. From a management perspective, judicious fertilization can increase both dry matter and forage quality simultaneously [19]. Ecologically, long-term cultivation of *Medicago sativa* L. has been shown to improve aggregate porosity, invigorate microbial diversity, and promote soil organic carbon accumulation [20,21]; in high-elevation alpine grasslands, infiltration and hydrological functioning are markedly enhanced [22]. Regarding the nitrogen cycle, symbiosis with rhizobia provides a key input via biological nitrogen fixation, typically around 200 kg N·ha^−1^·yr^−1^ [23,24]. As environmentally friendly inputs, nitrogen-fixing inoculants repeatedly increase the relative abundance of beneficial rhizosphere taxa and suppress potential pathogens, thereby improving rhizosphere ecology [25]. Nodulation and nitrogen fixation activity are enhanced, raising *Medicago sativa* L. nitrogen-use efficiency by 20–40% [26,27]. The advantages of seed coating have been documented under various stress conditions [28]: in Cu-contaminated soils, inoculation with Cu-tolerant Sinorhizobium meliloti elevates antioxidant enzyme activities and leaf nutrient contents, benefiting growth [29]; in saline–alkali soils, combined inoculation with Rhizobium and plant growth-promoting rhizobacteria (PGPR) increases nodulation, yield, and quality while reshaping rhizosphere communities, with convergent evidence from field and greenhouse studies [30,31]. Co-inoculation with arbuscular mycorrhizal fungi further synergizes phosphorus and nitrogen accumulation and biomass production [32]. Consequently, manipulating the rhizosphere micro-ecology with rhizobia or PGPR is considered both feasible and potentially effective for stabilizing and increasing yields in cold and arid regions such as the Qaidam Basin.

A critical knowledge gap remains, however: in the hydrothermally sensitive Qaidam Basin, the pathways and integrated effects through which different rhizobial seed-coating doses influence *Medicago sativa* L. yield—via changes in soil physicochemical properties and rhizosphere bacterial communities—are not well understood. To address this gap, we conducted a field experiment in the Delingha region using the cultivar ‘Beilin 201’. An uninoculated control and four inoculant doses—E1 (0.75 g·m^−2^), E2 (1.50 g·m^−2^), E3 (2.24 g·m^−2^), and E4 (3.00 g·m^−2^)—were established as a treatment gradient. Effects were evaluated against three hypotheses: (i) inoculation would significantly alter rhizosphere bacterial source composition, diversity, and community stability; (ii) inoculation would reconfigure the rhizosphere bacterial molecular ecological network; and (iii) diversity and network stability would affect yield directly or indirectly by modulating nutrient acquisition and interspecific interactions. By testing these hypotheses, our aim is to provide a scientific basis and practical guidance for optimizing rhizobial inoculant use in *Medicago sativa* L. production under cold and arid conditions, thereby improving land-use efficiency and delivering ecological and economic benefits.

## 2. Materials and Methods

### 2.1. Study Area, Experimental Design, and Field Management

**Figure 1:**
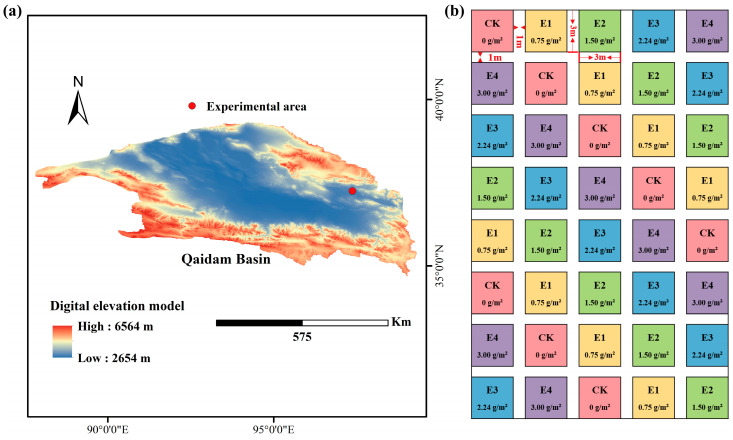
Geographic location of the experimental site and plot layout. (**a**) Study area. (**b**) Plot layout. Note: Data source for panel (**a**) basemap: National Cryosphere Desert Data Center (http://www.ncdc.ac.cn). CK, untreated control; E1 (0.75 g/m^2^), E2 (1.50 g/m^2^), E3 (2.24 g/m^2^), E4 (3.00 g/m^2^); *n* = 8 per treatment.

The field experiment was initiated on 1 May 2023, and continued for one growing season until *Medicago sativa* L. reached physiological maturity in October 2023. At physiological maturity, five sampling points were randomly selected along the diagonal of each plot (2800 m a.s.l.; mean annual temperature 2.8 °C; mean annual precipitation 181.8 mm; Figure 1a). Before treatment application, composite topsoil samples were collected from the experimental field to characterize the baseline soil conditions (Table S1). The site is flat, of inherently low fertility, and classified as sandy soil typical of cold and arid regions [33]. A single-factor randomized complete block design was adopted, with a seeding rate of 2000 g per 0.067 ha. Based on seed mass proportion, five rhizobial seed-coating treatments were established: CK (untreated control), E1 (0.75 g·m^−2^), E2 (1.50 g·m^−2^), E3 (2.24 g·m^−2^), and E4 (3.00 g·m^−2^). Each treatment had eight replicates (40 plots in total; 3 m × 3 m; Figure 1b). The test material was the *Medicago sativa* L. cultivar ‘Beilin 201’ (approved by the Ministry of Agriculture and Rural Affairs, Announcement No. 52).

The seed-coating product used in this study was a rhizobial-based composite microbial inoculant, primarily composed of rhizobia and supplemented with phosphate-solubilizing bacteria and Bacillus subtilis. The inoculant contained ≥5 × 10^8^ CFU·mL^−1^ viable cells and ≥6% total nutrients (N + P_2_O_5_ + K_2_O). The seed coating content and ratio were strictly adhered to according to industry standards (NY/T 798-2006) and previous optimization studies [33,34]. Prior to sowing, the field was plowed, and basal fertilizers were applied: 350 kg·ha^−1^ of organic fertilizer and 375 kg·ha^−1^ of compound fertilizer (N:P_2_O_5_:K_2_O = 25:12:5). According to the assigned treatment, the inoculant was thoroughly mixed with the seed to ensure uniform adhesion, air-dried in the shade, and sown within 12 h in furrows at a depth of 3–4 cm with 30 cm row spacing, followed by rolling. Weeding was performed twice during the seedling stage. Throughout the growing season, supplemental irrigation was provided via a motorized sprinkler, and uniform field management was maintained across all treatments.

### 2.2. Soil Sample Collection

At physiological maturity in October 2023, five points were randomly selected along the diagonal of each plot. At each point, three soil cores (0–15 cm depth; 38 mm diameter) were collected using a root auger. After gently shaking to remove loosely adhering soil, tightly adhering rhizosphere soil was brushed into sterile bags for physicochemical analyses; intact root systems with attached rhizosphere soil were bagged separately. Samples were transported to the laboratory at −20 °C by vehicle and promptly stored at −80 °C pending rhizosphere soil extraction.

Fresh roots with attached rhizosphere soil were suspended in sterile PBS buffer containing 0.1% Tween-80, vigorously shaken, and then sonicated for 10 min under low-temperature conditions to facilitate rhizosphere soil detachment while minimizing DNA degradation. The same procedure was applied consistently to all samples [35]. The supernatant was transferred to a new tube, and the procedure was repeated twice. The combined suspensions were centrifuged at 6000 rpm for 5 min to collect the pellet [33]. The resulting rhizosphere soil was freeze-dried at −40 °C for ≥12 h, sealed, and stored at −80 °C until DNA extraction.

### 2.3. DNA Extraction, PCR Amplification, Sequencing, and Sequence Analysis

Total DNA was extracted from 0.25 g of rhizosphere soil using the MoBio PowerSoil DNA Isolation Kit (QIAGEN Inc., Germantown, MD, USA). The V4 region of the 16S rRNA gene was amplified with primers 515F and 806R, and amplicons were verified by electrophoresis on 2% agarose gels, excised, and purified using the AxyPrep DNA Gel Extraction Kit (Axygen Biosciences, Union City, CA, USA). Paired-end (PE250) libraries were prepared according to Illumina standard protocols and sequenced on an Illumina MiSeq platform (Shanghai LingEn Biotechnology Co., Ltd., Shanghai, China). Sequences shorter than 50 bp were removed. Paired-end reads were merged only when the overlap length was ≥10 bp and the maximum mismatch rate was ≤0.2, followed by chimera removal. High-quality sequences were denoised using UNOISE and resolved into amplicon sequence variants (ASVs). The resulting ASV abundance table was used for subsequent analyses of community structure, diversity, and network construction. Prior to network analysis, ASVs with sufficient occurrence frequency across biological replicates within each treatment were retained to reduce spurious correlations caused by rare taxa [36].

### 2.4. Plant and Soil Measurements and Methods

At physiological maturity in October 2023, eight 50-cm harvest strips were established per plot. Whole plants within each strip were clipped and oven-dried to determine yield: samples were first heated at 105 °C for 30 min to inactivate enzymes, then dried at 65 °C to constant mass for dry matter conversion. Soil pH was measured at a 1:2.5 soil to water ratio using a PHS-3C pH meter. Total nitrogen (TN) was determined by the semi-micro Kjeldahl method [37]; total phosphorus (TP) by continuous-flow ammonium-molybdate spectrophotometry [38]; and total potassium (TK) after water-bath digestion by atomic fluorescence spectrometry [39]. Soil organic matter (OM) was quantified by the external heating K_2_Cr_2_O_7_–H_2_SO_4_ wet oxidation method [40]. Nitrate-N (NO_3_^−^–N) was measured by UV spectrophotometry [41], and ammonium-N (NH_4_^+^–N) by indophenol-blue colorimetry [42].

### 2.5. Statistical Analysis

Prior to statistical analysis, yield, soil physicochemical variables, microbial diversity indices, and network parameters were examined for normality and homoscedasticity. When data satisfied normality and equal-variance assumptions, independent-samples *t*-tests or one-way ANOVA were applied with Tukey HSD for post hoc comparisons; otherwise, the nonparametric Kruskal–Wallis test was used. All *p*-values were adjusted using the Bonferroni method [43].

Rhizosphere bacterial source contributions were inferred with FEAST, yielding the proportion of each target community derived from each source [44,45]. ASV set intersections were visualized using UpSetR [46,47]. Community structure was ordinated by PCoA and NMDS based on Bray–Curtis dissimilarities in the vegan package, with differences assessed by PERMANOVA and ANOSIM [48]; *p*-values were Bonferroni-corrected when evaluating among-treatment differences [49]. Community stability was quantified as the reciprocal of the average variation degree (RAVD), with higher RAVD indicating greater stability [50].

Molecular ecological networks were constructed under the Random Matrix Theory (RMT) framework using treatment-specific ASV abundance matrices. Networks for CK, E1, E2, E3, and E4 were built separately from eight independent biological replicates per treatment, without physical pooling of samples. Associations with |r| > 0.8 and FDR-adjusted *p* < 0.05 were retained for network construction, and the resulting networks were used for subsequent topological analyses [51,52]. Sample-specific subnetworks were extracted from the meta-network [53], and standard topological descriptors were calculated with the subgraph function in *igraph* [54]. Network dissimilarity indices were then used to assess among-treatment differences, with values approaching 1 indicating greater divergence [55]. Network robustness was defined as the proportion of species remaining after random removal of 50% of the nodes [56,57], and vulnerability was quantified as each node’s relative contribution to global efficiency [56]. Cohesion metrics were partitioned into positive cohesion and absolute negative cohesion, which quantify the relative strength of positive and negative co-occurrence associations, respectively. Because these associations are correlation-based, they should not be interpreted as direct evidence of cooperation or competition among taxa; reporting negative cohesion in absolute terms facilitates direct comparison with positive cohesion. Total cohesion was used to indicate the overall level of network connectedness [58]. In analyses of microbial network complexity, five key indicators were used. The number of nodes reflects the total count of biological entities in the network and serves as a proxy for biodiversity; the number of edges denotes the set of pairwise interactions, with higher values indicating denser connectivity; the average degree captures the mean number of connections per node and may imply more intricate interrelationships; the global clustering coefficient quantifies the prevalence of locally dense substructures, indicative of potential functional guilds and more elaborate organizational architecture; and modularity measures the extent of hierarchical partitioning and compartmentalization. All metrics were Z-score standardized to eliminate scale effects, and indicator weights were objectively determined via the entropy-weight method [59]. A weighted summation then yielded a composite network complexity index, providing an integrated depiction of structural and functional complexity [60,61]. For network stability, four indicators were selected. The ratio Positive/|Negative| compares the influence of cooperative linkages (positive cohesion) to that of competitive linkages (absolute negative cohesion), with larger values indicating a higher relative weight of cooperation [62,63]; total cohesion represents the overall strength of community connectedness, with larger values signifying tighter integration [58]; robustness reflects resistance to disintegration (larger values denote a lower propensity to collapse) [64]; and vulnerability quantifies sensitivity to the most destructive single-node failure [65]. To construct a composite network stability index, we used Positive/|Negative|, total cohesion, robustness, and the reciprocal of the maximum vulnerability (to harmonize indicator directions so that larger values uniformly imply greater stability). All indicators were Z-score standardized, weighted by the entropy-weight method [59], and aggregated by weighted summation [66].

To identify the key drivers of *Medicago sativa* yield, variance inflation factors (VIFs) were calculated to exclude collinear predictors (VIF > 10). Because the experiment was conducted at a single field site, field was not included as a random effect. Treatment was considered a fixed effect. Where mixed-effects models were used, the experimental block or replicate structure was included as a random intercept to account for the randomized complete block design. All-subset model selection was performed using the MuMIn package, and the relative contributions of selected predictors to explained yield variation were further partitioned using the glmm.hp package [67]. Stratified decomposition was conducted using the glmm.hp package to estimate the relative contributions of each predictor to the explained variance (marginal R^2^) [68]. Data visualization was performed using the ggplot2 package. The selected variables were then incorporated into a piecewise structural equation model (SEM) using the piecewiseSEM package [69]. The SEM examined the mechanisms through which soil and microbial factors regulate yield via direct or indirect pathways under the application of rhizobial inoculants. The model’s suitability was confirmed through non-significant model fit tests (*p* > 0.05), and the optimal model was selected based on Chi-Squared and Fisher’s C values, indicating its superior explanatory power [70]. All analyses were conducted in R 4.4.1.

## 3. Results and Analysis

### 3.1. Effects of Rhizobial Inoculation on Medicago sativa *L.* Yield and Rhizosphere Soil Physicochemical Properties

**Figure 2:**
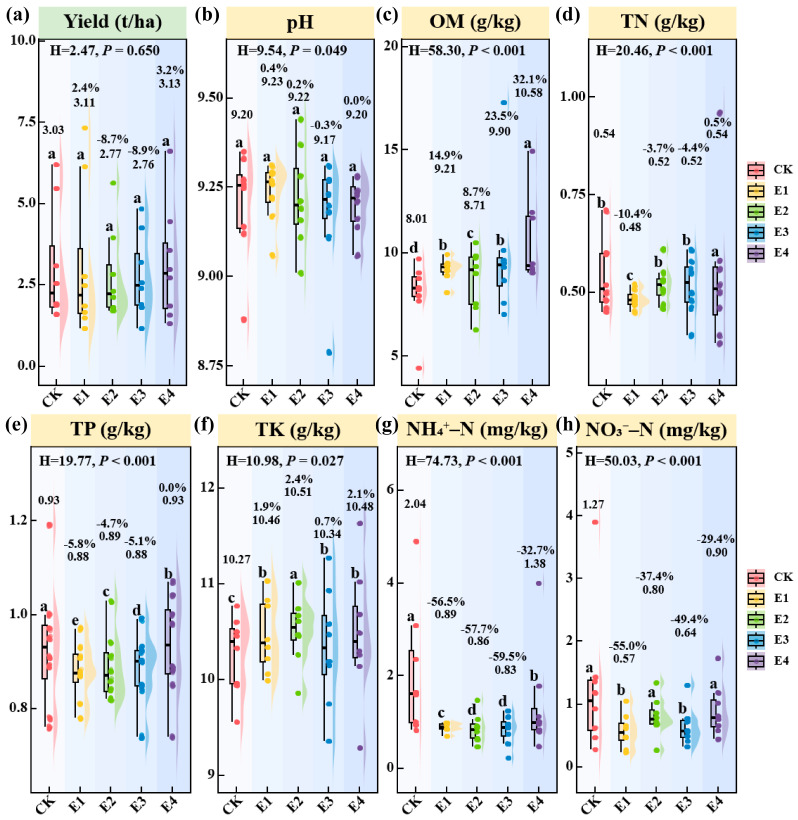
Effects of rhizobial inoculation on *Medicago sativa* L. yield and rhizosphere soil physicochemical properties. (**a**) *Medicago sativa* L. yield. (**b**–**h**) Rhizosphere soil physicochemical properties. Note: OM, soil organic matter; TN, total nitrogen; TP, total phosphorus; TK, total potassium; NH_4_^+^–N, ammonium-N; NO_3_^−^–N, nitrate-N. Different lowercase letters indicate significant differences among treatments. CK, untreated control; E1, 0.75 g·m^−2^; E2, 1.50 g·m^−2^; E3, 2.24 g·m^−2^; E4, 3.00 g·m^−2^ (*n* = 8 per treatment).

The composite microbial inoculant did not significantly increase *Medicago sativa* L. yield under the present short-term field conditions (Figure 2a). Yield was generally maintained across treatments, with only modest increases observed under E1 and E4, corresponding to gains of 2.4% and 3.2%, respectively. Soil organic matter (OM) increased significantly with inoculant rate (E4 > E3 > E2 > E1 > CK; *p* < 0.001; Figure 2c), whereas ammonium-N (NH_4_^+^–N) and nitrate-N (NO_3_^−^–N) decreased significantly (both *p* < 0.001; Figure 2g,h). The greatest reductions were observed for NH_4_^+^–N under E3 (−59.5%) and for NO_3_^−^–N under E1 (−55.0%). Soil pH remained essentially unchanged, consistent with the short-term resistance of alkaline buffering systems, while total N rebounded under higher-dose treatments (Figure 2b,d). Collectively, rhizobial inoculation may have enhanced biological nitrogen fixation and nutrient uptake, thereby increasing nitrogen assimilation by plants and associated microbes while promoting soil organic matter accumulation.

### 3.2. Effects of Rhizobial Inoculation on Rhizosphere Bacterial Community Composition

**Figure 3:**
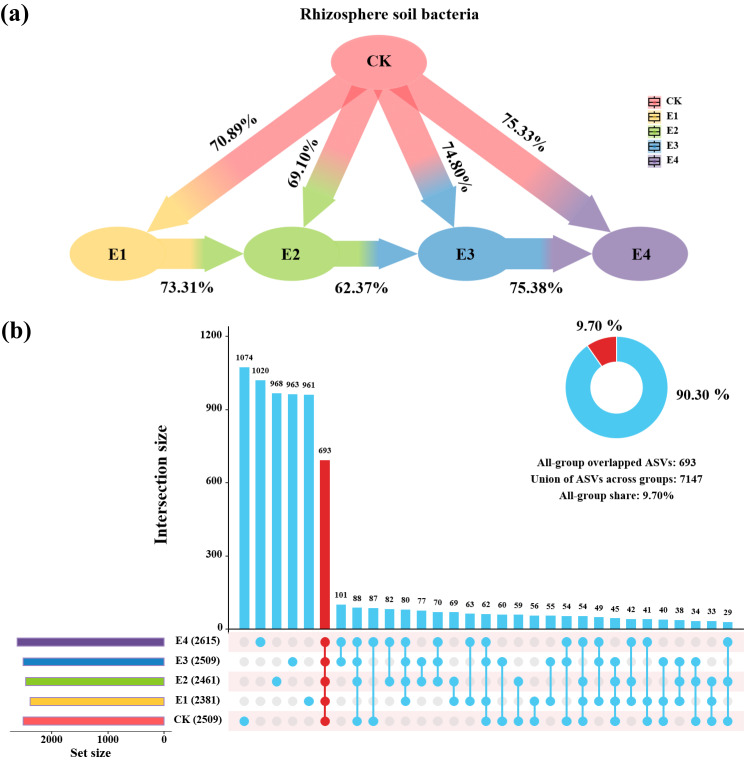
Rhizosphere bacterial source tracking and community composition. (**a**) Source tracking of rhizosphere bacteria (FEAST-based; percentages indicate the fraction of the Target community attributed to each Source). (**b**) UpSet plot of rhizosphere bacterial ASVs (top bars, intersection sizes for treatment combinations; left bars, total set size per treatment; blue dots and connecting lines, combinations included in each intersection). Note: CK, untreated control; E1, 0.75 g·m^−2^; E2, 1.50 g·m^−2^; E3, 2.24 g·m^−2^; E4, 3.00 g·m^−2^ (*n* = 8 per treatment).

Source tracking indicated that 70.89%, 69.10%, 74.80%, and 75.33% of the bacterial assemblages in E1, E2, E3, and E4, respectively, were derived from CK (Figure 3a). Moreover, approximately 73.31% of the E2 community was attributable to E1, while 62.37% (E3) and 75.38% (E4) could be traced to the adjacent treatment. UpSet analysis further revealed a shared set of 693 ASVs among all treatments (Figure 3b), representing 9.7% of the total ASVs. With increasing inoculant dose, ASV richness showed a decrease-then-increase pattern: E1 and E2 contained 2381 and 2461 ASVs, respectively; E3 matched CK with 2509 ASVs; and E4 was highest at 2615 ASVs. At the phylum level (Supplementary Figure S1), *Proteobacteria* and *Actinobacteriota* were dominant; at the genus level, “Others” (unclassified) and *Pseudomonas* accounted for large proportions, whereas rhizobial-affiliated taxa were present but at overall low relative abundances.

### 3.3. Effects of Rhizobial Inoculation on Rhizosphere Bacterial Diversity and Community Stability

**Figure 4:**
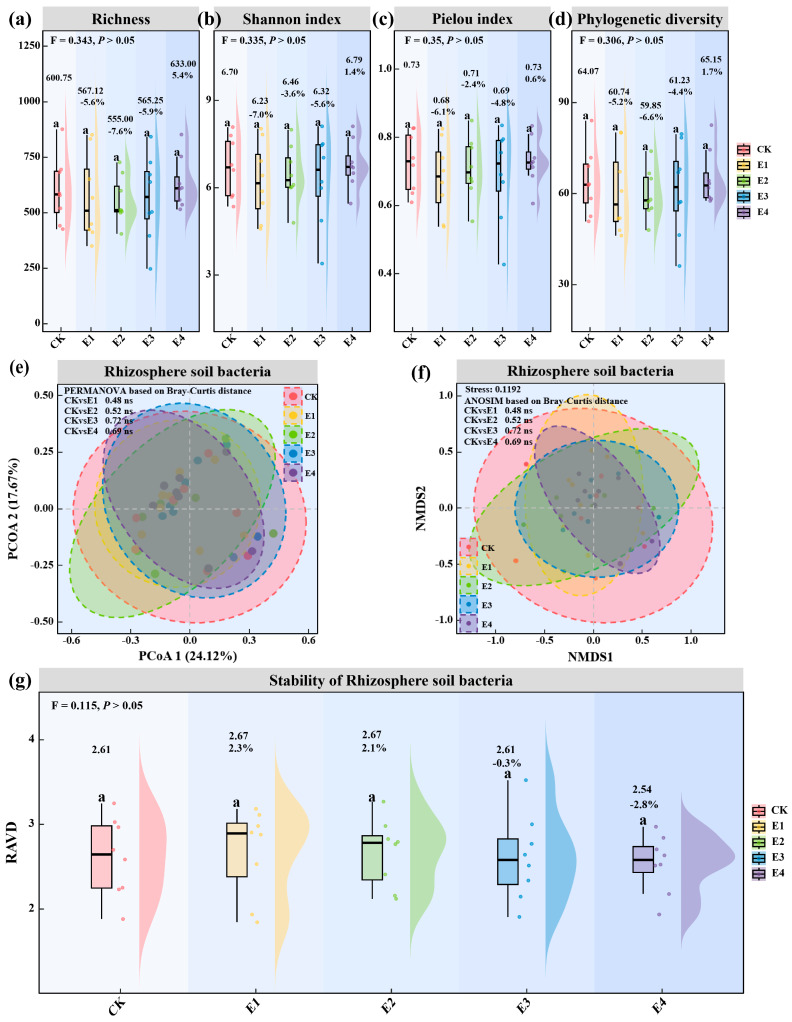
(**a**) Richness; (**b**) Shannon index; (**c**) Pielou index; (**d**) Phylogenetic diversity; (**e**) principal coordinate analysis (PCoA); (**f**) non-metric multidimensional scaling (NMDS); (**g**) community stability of rhizosphere soil bacteria (based on RAVD). Note: CK, untreated control; E1 (0.75 g/m^2^), E2 (1.50 g/m^2^), E3 (2.24 g/m^2^), E4 (3.00 g/m^2^) (*n* = 8 per treatment). The same lowercase letters indicate no significant differences among treatments (*p* > 0.05).

Across treatments, α-diversity did not differ significantly (*p* > 0.05; Figure 4a–d), although the high-dose treatment E4 showed slight increases in richness, Shannon index, Pielou’s evenness, and phylogenetic diversity. Principal coordinate analysis (PCoA) indicated that the first two axes explained 24.12% and 17.67% of the total variation, respectively (*p* > 0.05; Figure 4e), with no evident separation among communities. Non-metric multidimensional scaling (NMDS) and ANOSIM corroborated these patterns, revealing no significant between-treatment differences in community structure (*p* > 0.05; Figure 4f).

RAVD—where higher values denote greater community stability—was modestly elevated under E1 and E2 (+2.3% and +2.1%), but decreased under E4 (−2.8%); however, differences among treatments were not significant (*p* > 0.05; Figure 4g). Overall, rhizobial seed coating exerted limited, dose-dependent influences on stability, with minimal net change in α-diversity and community robustness across the tested gradient.

### 3.4. Effects of Rhizobial Inoculation on Rhizosphere Bacterial Networks

**Figure 5:**
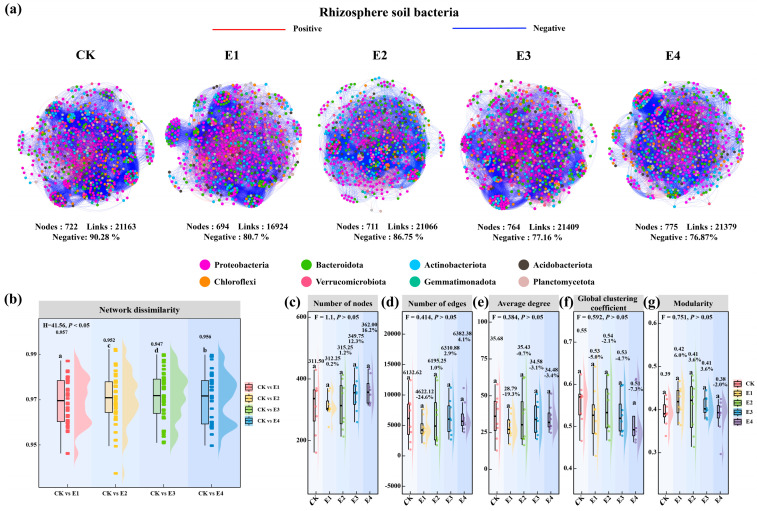
Effects of rhizobial inoculation on rhizosphere bacterial networks. (**a**) Rhizosphere bacterial co-occurrence networks (annotated at the phylum level; node size denotes degree; edge thickness denotes weight; red edges indicate positive correlations, blue edges indicate negative correlations); (**b**) Network dissimilarity of rhizosphere bacterial communities; (**c**) Number of nodes; (**d**) Number of edges; (**e**) Average degree; (**f**) Global clustering coefficient; (**g**) Modularity. Note: CK, untreated control; E1, 0.75 g·m^−2^; E2, 1.50 g·m^−2^; E3, 2.24 g·m^−2^; E4, 3.00 g·m^−2^ (*n* = 8 per treatment). The same lowercase letters indicate no significant differences among treatments (*p* > 0.05).

Rhizobial seed coating markedly reconfigured the rhizosphere bacterial co-occurrence networks (Figure 5a; Supplementary Table S2). As the inoculant application rate increased, the proportion of negative associations decreased, whereas the proportion of positive associations increased. These changes indicate that the correlation structure of the rhizosphere bacterial network was altered, but they do not necessarily reflect direct ecological interactions among taxa: negative edges were most prevalent in CK (90.28%) and least prevalent in E4 (76.87%). Network dissimilarity relative to CK approached 1 across treatments (Figure 5b), indicating pronounced structural perturbation, with the greatest dissimilarity observed between CK and the low-dose E1 (0.957).

In terms of topology (Figure 5c–g), both the number of nodes and the number of edges increased with dose; in E4, nodes and edges were higher by 16.2% and 4.1%, respectively. By contrast, mean degree was highest in CK and decreased with increasing dose. The global clustering coefficient likewise peaked in CK and was lowest in E4 (−7.3%), while modularity was slightly reduced under E3 and E4—together indicating a more diffuse network at higher doses with attenuated local clustering.

**Figure 6:**
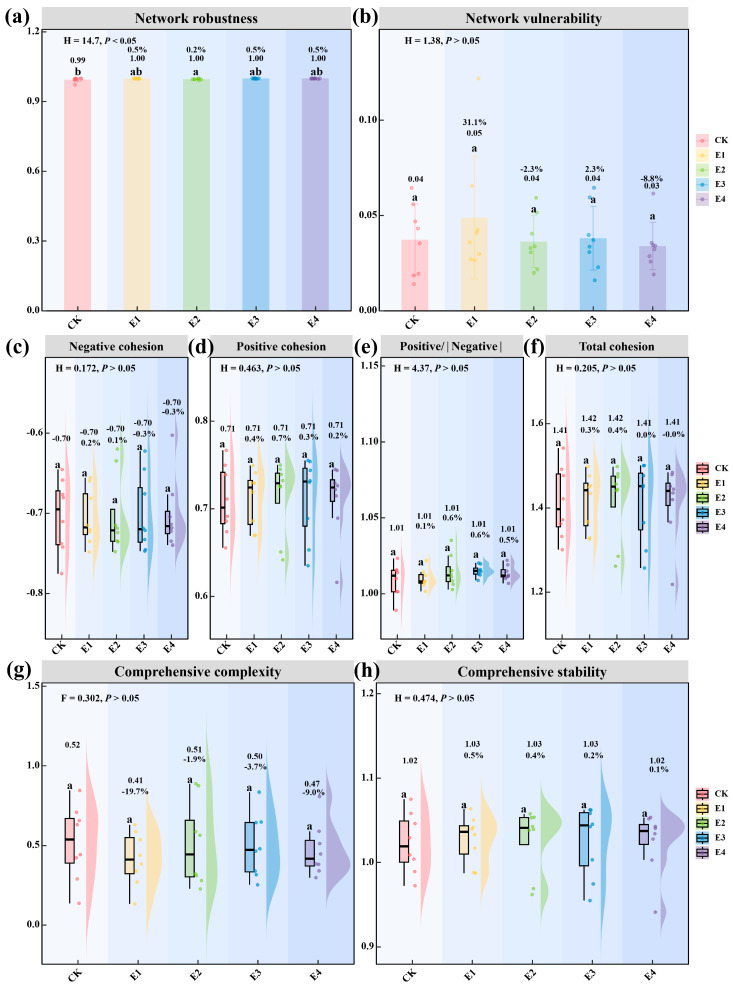
Effects of rhizobia on the network stability of rhizosphere soil bacteria. (**a**) Network robustness of rhizosphere soil bacteria (robustness quantified as the proportion of taxa remaining after randomly removing 50% of taxa from each empirical molecular ecological network, MEN); (**b**) Vulnerability of rhizosphere soil bacteria; (**c**) Negative cohesion; (**d**) Positive cohesion; (**e**) Positive/∣Negative∣ cohesion; (**f**) Total cohesion; (**g**) Composite network complexity; (**h**) Composite network stability. Note: CK, untreated control; E1 (0.75 g/m^2^), E2 (1.50 g/m^2^), E3 (2.24 g/m^2^), E4 (3.00 g/m^2^) (*n* = 8 per treatment). Different lowercase letters indicate significant differences among treatments (*p* < 0.05).

Following rhizobial seed coating, the robustness of rhizosphere bacterial networks was generally enhanced relative to CK, with a statistically significant difference observed between CK and E2 (*p* < 0.05; Figure 6a). In terms of vulnerability, E1 exhibited the highest value, whereas E4 showed the lowest, representing a reduction of 8.8% (*p* > 0.05; Figure 6b). Overall, inoculation increased network dissimilarity and altered network topological properties, indicating a restructuring of rhizosphere bacterial network architecture.

Regarding cohesion, neither negative cohesion nor positive cohesion differed significantly among treatments (*p* > 0.05; Figure 6c–f), indicating that the intensities of competitive and cooperative interactions remained broadly consistent across treatments. Comprehensive complexity was highest in CK (Figure 6g), while E1 demonstrated the greatest comprehensive stability; however, these differences were not statistically significant (*p* > 0.05; Figure 6h). Collectively, these findings indicate that, under the present experimental conditions, rhizobial application exerted a limited influence on the overall stability of the rhizosphere bacterial network.

### 3.5. Key Drivers of Yield and Pathways of Influence

**Figure 7:**
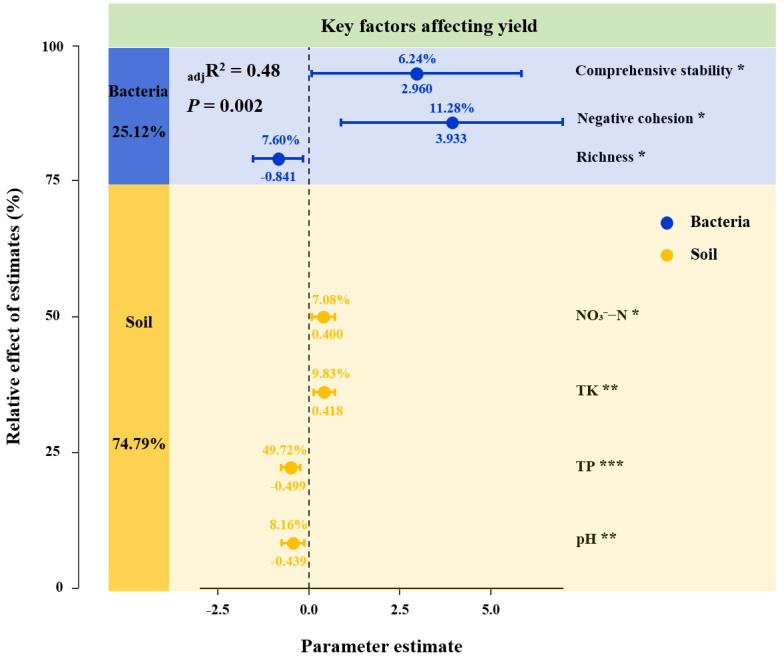
Relative contributions of key drivers of *Medicago sativa* L. yield. Note: Total phosphorus (TP); total potassium (TK); nitrate nitrogen (NO_3_^−^–N); * 0.01 < *p* < 0.05; ** 0.001 < *p* < 0.01; *** *p* < 0.001.

The mixed effects model revealed the key factors affecting yield under rhizobial inoculant application. In the microbial domain, richness (7.60%), negative cohesion (11.28%), and cmprehensive stability (6.24%), along with pH (8.16%), TP (49.72%), TK (9.83%), and NO_3_^−^–N (7.08%) in the soil domain, were identified as the dominant factors influencing yield (Figure 7; _adj_R^2^ = 0.48), with significant contributions (*p* = 0.002).

**Figure 8:**
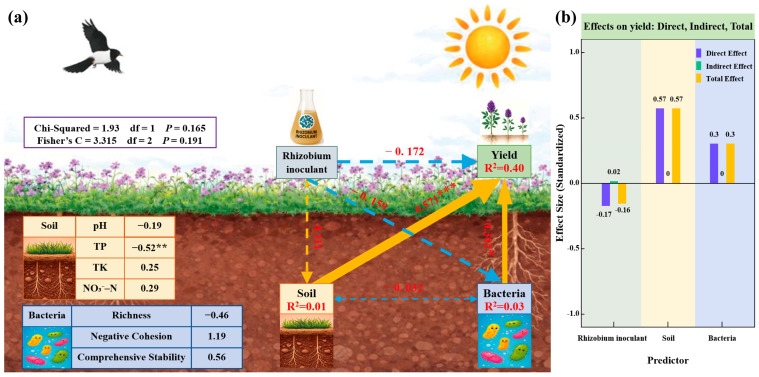
Direct and indirect pathways linking rhizobial inoculation, soil properties, microbial variables, and *Medicago sativa* L. yield. (**a**) Piecewise structural equation model. Values beside arrows are standardized path coefficients; solid and dashed arrows indicate significant and non-significant paths, respectively. (**b**) Standardized direct, indirect, and total effects on yield. TP, total phosphorus; TK, total potassium; NO_3_^−^–N, nitrate nitrogen. * *p* < 0.05, ** *p* < 0.01, *** *p* < 0.001.

A structural equation model (SEM) was constructed to clarify the regulatory pathway by which rhizobial inoculants (Rhizobium inoculant) affect *Medicago sativa* L. yield. The final model showed good fit (Chi-Squared = 1.93, df = 1, *p* = 0.165, Fisher’s C = 3.315, df = 2, *p* = 0.191; Figure 8a) and explained 40% of the variation in *Medicago sativa* L. yield (R^2^ = 0.4). Soil had a direct effect of 0.57 on yield (Figure 8b), microbial factors had a direct effect of 0.3 (Figure 8b), while rhizobial inoculant had a weak negative direct effect (−0.17; Figure 8b), with its primary impact being transmitted indirectly through the soil pathway. In summary, the increase in TK and pH, the decrease in TP and NO_3_^−^–N, the reduction in Richness, and the reorganization of the ecological network towards greater stability were identified as the key mechanisms by which rhizobial inoculants influence *Medicago sativa* L. yield.

## 4. Discussion

### 4.1. Rhizobial Inoculation Increases Medicago sativa *L.* Yield and Soil Organic Matter While Markedly Reducing Ammonium and Nitrate

As a canonical legume–rhizobium symbiosis, rhizobial seed coating of *Medicago sativa* L. has repeatedly been shown to promote nodulation, enhance yield, and improve nitrogen nutrition [31,71]. In the present short-term field experiment, the composite microbial inoculant did not significantly increase yield, although modest increases of 2.4% and 3.2% were observed under E1 and E4, respectively. These responses were accompanied by significant increases in soil organic matter and changes in mineral nitrogen pools. Soil pH remained essentially unchanged, consistent with the consensus that short-term biotic interventions exert limited influence on pH and that alkaline buffering constrains symbiotic responses [72,73]. By contrast, soil organic matter (OM) rose significantly with inoculant rate (E4: +32.1%), in agreement with reports that rhizobial inoculation augments OM and microbial activity and improves soil physicochemical properties [71,74]. At higher doses, total nitrogen (TN) converged toward the control, while nitrogen became more readily utilized by plants, a pattern aligned with frameworks in which plants regulate symbiotic nitrogen fixation according to mineral nitrogen supply and endogenous nitrogen demand [75]. Total potassium (TK) increased slightly, whereas total phosphorus (TP) declined slightly, echoing findings from long-term or mixed cropping systems in which inoculation more readily increases plant-available phosphorus while exerting smaller effects on potassium—suggesting divergence between short- and long-term responses [76,77]. Concomitantly, soil ammonium-N (NH_4_^+^–N) and nitrate-N (NO_3_^−^–N) decreased sharply across all coating treatments, with maximum reductions of 59.5% for NH_4_^+^–N (E3) and 55.0% for NO_3_^−^–N (E1). The significant reductions in NH_4_^+^–N and NO_3_^−^–N indicate substantial changes in the rhizosphere mineral nitrogen pool. The rhizobial inoculant may have altered the balance among plant nitrogen uptake, microbial immobilization, and nitrogen transformation processes, while biological nitrogen fixation may represent only one of several potential contributing pathways. Future studies should incorporate assessments of nodulation traits, nitrogenase activity, plant nitrogen accumulation, and functional genes involved in nitrification and mineralization to further elucidate the underlying mechanisms [78,79]. In sum, without appreciably altering pH in alkaline soils, rhizobial inoculation modestly increased *Medicago sativa* L. yield and substantially elevated OM while driving pronounced decreases in inorganic nitrogen; short-term responses of phosphorus and potassium were minor but may become more pronounced over longer timescales.

### 4.2. Rhizobial Inoculation Within the Tested Dose Gradient Did Not Significantly Alter the Medicago sativa *L.* Rhizosphere Bacterial Community

Multiple studies have shown that rhizobial inoculation can markedly enhance soil bacterial diversity and promote community turnover and adaptation [80]. Consistent with this, FEAST source tracking indicated that >69% of the communities under inoculated treatments were traceable to CK, with compositional continuity between adjacent doses—corroborating the view that edaphic and site factors often outweigh inoculation effects [45,81]. UpSet analysis further identified 693 ASVs shared across all five treatments (9.70%), aligning with reports that only a small subset of ASVs constitutes the rhizosphere core in crops [82,83]. Although rhizobial or PGPR seed coating is widely used to improve stress performance and nutrient use efficiency in legumes such as *Medicago sativa* L., shifts in overall α-diversity, β-diversity, and phylum- or genus-level structure are not invariably significant and are jointly modulated by inoculant strains, soil context, and stress regimes [84,85,86]. Field studies likewise report increased proportions of *Proteobacteria* and *Actinobacteriota* following biofertilizer use without pronounced community-level divergence [12]. In line with these observations, *Proteobacteria* remained consistently dominant at the phylum level in our study, and *Pseudomonas* was identified as a leading genus; both are common and functionally important rhizosphere groups with broad application potential across crops [81,87]. Across inoculant doses, α-diversity differences are non-significant, and communities do not separate distinctly; similarly, field and pot experiments often find that inoculation does not markedly perturb native rhizosphere assemblages, or its influence is smaller than that of tillage and site heterogeneity [88,89]. While inoculation may affect diversity, its magnitude appears co-regulated by application rate and soil environment [90]. With respect to stability, RAVD increased slightly under the low doses E1 and E2 but declined under the high dose E4, with no significant among-treatment differences overall—consistent with theories of soil microbial community stability and resistance to disturbance [91]. Collectively, inoculation imparted some compositional carryover relative to the control; however, within the present dose gradient, community stability and diversity were largely maintained, and the overarching community framework was not substantially rewritten.

### 4.3. Rhizobial Inoculation Significantly Reshapes the Rhizosphere Bacterial Ecological Network

RMT-based inference has proven effective for identifying microbial co-occurrence networks and diagnosing responses to environmental perturbations [56]; in between-group comparisons, this framework emphasizes network dissimilarity metrics to detect treatment effects [92]. In our study, network dissimilarity indices between each inoculated treatment and CK approached 1, indicating pronounced topological rewiring. With increasing inoculant dose, the proportion of negative (putatively competitive) edges declined, while the global clustering coefficient and modularity were depressed; by contrast, the numbers of nodes and edges increased. This configuration—strengthened overall connectedness coupled with weakened local modular organization—has been repeatedly documented in rhizosphere networks along fertilization, N management, and land use gradients [93,94,95]. Regarding stability, robustness increased after inoculation, and vulnerability decreased at higher doses, consistent with assessments showing that random loss of a few peripheral nodes has limited impact, whereas targeted removal of hubs markedly reduces connectivity and stability [96]. Cohesion, a metric quantifying community connectedness and linked to community dynamics, was applied to test dose effects [62]; neither negative nor positive cohesion differed significantly among treatments, indicating that the overall intensities of competition and cooperation remained relatively constant. Overall, network dissimilarity increased, whereas cohesion metrics remained largely stable. These results indicate that rhizobial inoculant treatment altered the network structure of the rhizosphere bacterial community.

### 4.4. Key Drivers and Pathways by Which Rhizobial Inoculants Influence Medicago sativa *L.* Yield

Phosphorus supply has long been considered a key limiting factor for the stable production of leguminous forage crops like *Medicago sativa* L. Proper phosphorus application can significantly increase the aboveground biomass, forage quality, and regrowth ability of *Medicago sativa* L., while phosphorus deficiency leads to slow growth, reduced nitrogen fixation efficiency, and significant yield loss [97,98,99,100]. Consistent with this, in the key drivers of *Medicago sativa* yield in this study, TP had a highly significant impact on yield (49.72%), followed by TK (9.83%). The SEM indicates that soil factors have a direct positive effect on yield (0.57). Mechanistically, through symbiosis with rhizobia, *Medicago sativa* L. is continuously supplied with nitrogen from biological nitrogen fixation, promoting protein accumulation and increasing biomass. Dominant symbiotic combinations are often observed to significantly improve yield and alleviate environmental stress [29,101,102]. The SEM provided a more nuanced interpretation of the inoculant effect. After soil and microbial variables were included in the model, the direct pathway from inoculant application rate to yield was weakly negative (−0.17), whereas the indirect pathway mediated by soil properties was positive. This result indicates that, after accounting for changes in soil properties, the inoculant did not directly increase yield within a single growing season. One possible explanation is that rhizobial inoculation initially altered nutrient allocation, rhizosphere resource competition, or microbial community adjustment, thereby weakening its immediate direct contribution to aboveground biomass. Meanwhile, the positive soil-mediated pathway suggests that the main benefit of inoculation was expressed more through changes in soil nutrient status than through a direct stimulation of yield. As a key biological foundation for grassland productivity, nutrient cycling, and ecological stability, the structure and interaction network of soil microbial communities have been repeatedly confirmed [103,104,105]. The topological features of their co-occurrence network are often used to characterize the system’s ability to maintain function under environmental fluctuations [105,106]. In this study, negative cohesion (11.28%) and comprehensive stability (6.24%) had significant effects on yield, and SEM showed that microbial factors have a positive direct effect on yield (0.3). In summary, the increase in TK and pH, the decrease in TP and NO_3_^−^–N, the reduction in richness, and the reorganization of the ecological network towards greater stability were identified as the key mechanisms by which rhizobial inoculants influence *Medicago sativa* L. yield. The differences between different treatment groups in the experiment should be attributed to the varying levels of rhizobial inoculation, and these differences were compared under a fixed and locally relevant fertilization background.

## 5. Conclusions

Under the cold and arid field conditions of Delingha in the Qaidam Basin on the Qinghai–Tibet Plateau, it has been confirmed that dose-dependent rhizobial seed coating can sustain stable *Medicago sativa* L. production. With increasing inoculant dose, soil organic matter is significantly increased, while ammonium and nitrate nitrogen are reduced. Meanwhile, the α-diversity, β-diversity, and overall structure of the rhizosphere bacterial community remain stable, but the ecological network is reshaped, with weakened negative correlations, increased nodes and edges, and improved network robustness. The mixed effects model revealed the impact of the soil and microbial domains on yield, and structural equation modeling showed that soil and microbial factors influence yield, with the inoculant dose primarily exerting an indirect positive effect through the soil pathway. Overall, this study provides field-based evidence that rhizobial seed coating can alter soil nutrient status and rhizosphere bacterial co-occurrence patterns in *Medicago sativa* L. production systems. However, longer-term and multi-site studies are required to determine whether these short-term responses translate into stable yield benefits or broader ecosystem-level effects on the Qinghai–Tibet Plateau.

## Data Availability

The original data presented in the study are openly available in the NCBI database with the study accession number PRJNA1353582 that are publicly accessible at https://ncbi.nlm.nih.gov/sra, accessed on 1 November 2025.

## Supplementary Materials

The following supporting information can be downloaded at: https://www.mdpi.com/article/10.3390/microorganisms14071427/s1, Figure S1. Rhizosphere soil bacteria at the phylum and genus levels (the numeric labels at the phylum and genus levels in the figure only show taxa with relative abundance > 1%). Table S1. Basic chemical properties of the plow layer soil for each treatment prior to sowing. Table S2. Rhizosphere soil bacterial network topology parameters.
