# Supplementary material for: Rhizobial Inoculation Improves Soil Properties and Microbial Network Stability to Support Medicago sativa L. Production in Cold Arid Regions
Source: Microorganisms. 2026 Jun 30;14(7):1427. doi: 10.3390/microorganisms14071427 (PMC13413457; doi:10.3390/microorganisms14071427)
Supplement: Supplementary file 1 [file microorganisms-14-01427-s001.zip › microorganisms-4316392-supplementary.pdf]

Supplementary materials for

Rhizobial Inoculation Improves Soil Properties and Microbial Network Stability to Support *Medicago sativa* L. Production in Cold Arid Regions

Qianqian Zhao [1], Xin Jin[1], Chengti Xu [2,3,4] , Guangxin Lu [1,*] and Haijuan Zhang [5,*]

[1] College of Forestry and Grassland, Qinghai University,
Xining 810016, China;
13141752025@163.com (Q.Z.); 18894310895@163.com (X.J.)
[2] Northwest Key Laboratory of Cultivated Land Conservation and Marginal Land Improvement, Ministry of Agriculture and Rural Affairs, Delingha 817000, China; xchti@163.com
[3] College of Animal Husbandry and Veterinary Sciences, Qinghai University, Xining 810003, China
[4] National Grass Varieties Regional Test Station, Delingha 817000, China
[5] Qinghai Provincial Key Laboratory of Plateau Climate Change and Corresponding Ecological and Environmental Effects, Qinghai Institute of Technology, Xining 810016, China
* Correspondence: lugx74@163.com (G.L.); hjzhang@qhit.edu.cn (H.Z.);
Tel.: +86-15509714323 (G.L.); +86-13897216290 (H.Z.)

Table S1 Basic chemical properties of the plow layer soil for each treatment prior to sowing

Test items	CK	E1	E2	E3	E4
pH/1:2.5	8.22±0.18[a]	8.12±0.33[a]	8.21±0.13[a]	8.18±0.21[a]	8.16±0.24[a]
Organic matter in soil/%	3.12±0.15[a]	3.13±0.12[a]	2.78±0.5[a]	2.95±0.32[a]	3.06±0.21[a]
Total phosphorus in soil/(mg·kg^{-1})	610.33±59.81[a]	612.33±50.58[a]	612.67±65.62[a]	611.67±55.24[a]	613.00±57.46[a]
Total nitrogen in soil/(mg·kg^{-1})	677.67±93.22[a]	688.33±68.54[a]	687±23.07[a]	681.33±45.62[a]	690.00±52.31[a]
Nitrate nitrogen in soil/(mg·kg^{-1})	12.97±1.51[a]	12.62±0.9[a]	11.43±0.59[a]	12.08±0.76[a]	11.86±0.81[a]
Ammonium nitrogen in soil/(mg·kg^{-1})	0.92±0.04[a]	0.61±0.05[a]	0.78±0.11[a]	0.72±0.08[a]	0.83±0.10[a]
Total potassium in soil/(g·kg^{-1})	16.45±9.87[a]	16.76±10.78[a]	16.78±11.34[a]	16.82±10.95[a]	16.70±11.12[a]

Note: Values conforming to a normal distribution are presented as "mean ± standard deviation". The same lowercase letters indicate no significant differences among treatments (one-way ANOVA and Tukey's HSD test, $p < 0.05$).

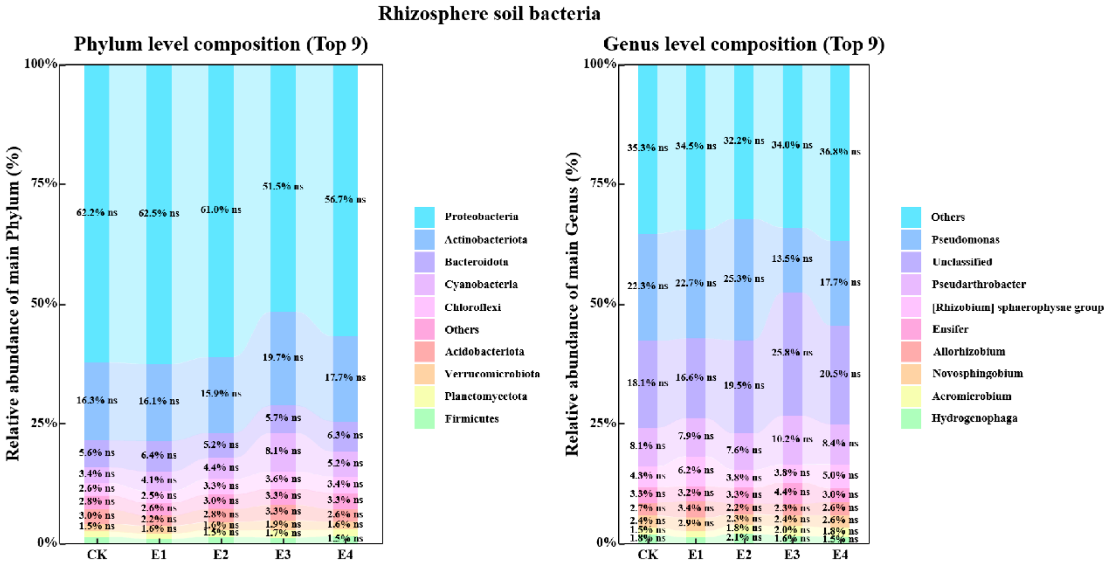

Figure S1. Rhizosphere soil bacteria at the phylum and genus levels (the numeric labels at the phylum and genus levels in the figure show only taxa with relative abundance > 1%). Note: CK, untreated control; E1（0.75 g/m^2）、E2（1.50 g/m^2）、E3（2.24 g/m^2）、E4（3.00 g/m^2）(n = 8 per treatment). Different lowercase letters indicate significant differences among treatments, ^{ns} $p > 0.05$.

Table S2.Rizosphere soil bacteria network topology parameters

Networks	Iten	Rizosphere soil bacteria				
		CK	E1	E2	E3	E4
Empirical networks	Total nodes	722	694	711	764	775
	Total links	21163	16924	21066	21409	21370
	Average degree (avgK)	58.62	48.77	59.26	59.05	55.17
	Density (D)	0.081	0.070	0.083	0.073	0.071
	Average clustering coefficient (avgCC)	0.558	0.524	0.535	0.512	0.519
	modularity	0.446	0.382	0.428	0.419	0.394
Random networks	Average clustering coefficient (avgCC)	0.042***	0.041***	0.040***	0.040***	0.039***
	modularity	0.446***	0.382***	0.428***	0.419***	0.393***

Note: CK, untreated control; E1（0.75 g/m²）、E2（1.50 g/m²）、E3（2.24 g/m²）、E4（3.00 g/m²）(n = 8 per treatment). Significance level of the metrics between empirical and random networks，****p* ＜ 0.001。
